# Effects of temperature gradient on functional fruit traits: an elevation-for-temperature approach

**DOI:** 10.1186/s12862-024-02271-w

**Published:** 2024-07-09

**Authors:** Laura Gómez-Devia, Omer Nevo

**Affiliations:** 1 German Centre for Integrative Biodiversity Research (iDiv) , Halle-Jena-Leipzig, Germany; 2 Global Change Research Group, Mediterranean Institute for Advanced Studies (IMEDEA, CSIC-UIB), Esporles, Spain; 3 Technische Universität Dresden, Dresden, Germany; 4 Institute of Biodiversity, Friedrich Schiller University Jena, Jena, Germany

**Keywords:** Climate change, Functional traits, Temperature, Frugivory, Global warming, Seed dispersal

## Abstract

Fruit traits mediate animal-plant interactions and have to a large degree evolved to match the sensory capacities and morphology of their respective dispersers. At the same time, fruit traits are affected by local environmental factors, which may affect frugivore-plant trait match. Temperature has been identified as a major factor with a strong effect on the development of fruits, which is of serious concern because of the rising threat of global warming. Nonetheless, this primarily originates from studies on domesticated cultivars in often controlled environments. Little is known on the effect of rising temperatures on fruit traits of wild species and the implications this could have to seed dispersal networks, including downstream consequences to biodiversity and ecosystem functioning. In a case study of five plant species from eastern Madagascar, we addressed this using the elevation-for-temperature approach and examined whether a temperature gradient is systematically associated with variation in fruit traits relevant for animal foraging and fruit selection. We sampled across a gradient representing a temperature gradient of 1.5–2.6 °C, corresponding to IPCC projections. The results showed that in most cases there was no significant effect of temperature on the traits evaluated, although some species showed different effects, particularly fruit chemical profiles. This suggests that in these species warming within this range alone is not likely to drive substantial changes in dispersal networks. While no systemic effects were found, the results also indicate that the effect of temperature on fruit traits differs across species and may lead to mismatches in specific animal-plant interactions.

**Supplementary Information:**

The online version contains supplementary material available at 10.1186/s12862-024-02271-w.

## Introduction

Many tropical plants rely on animals for seed dispersal [1, 2] in a mutualistic interaction in which frugivorous animals obtain nutritious food and plants disperse their seeds [3, 4]. Frugivores differ in their senses, fruit handling capacities, foraging behavior, and preferences [5, 6] and fruit traits have to a large degree evolved to match the different sensory capacities and morphologies of their respective frugivores [7, 8]. Therefore, trait matching has been recognized as an important mechanism mediating the structure of seed dispersal networks [9, 10]. Specifically, traits such as scent [6, 11] color [7, 12], size [13], hardness [14] and nutritional content [15] have been shown to play a major role in frugivore foraging and food selection.

At the same time, fruit traits are liable to environmental factors [4, 16, 17]. Work primarily on cultivated species like mangos, avocados, strawberries, bananas and grapes, revealed that temperature in particular is a key factor affecting fruit development, together with humidity, UV-light intensity, day/night temperature variance, precipitation and soil type, although to a lesser degree [16, 18]. During fruit development higher temperatures are associated with changes in fruits at the morphological, physiological and biochemical level [16], but the responses of specific fruit traits to temperature are mixed [17, 18]. In general, it has been observed that higher temperatures decrease fruit sugar content, size and hardness of fruits [18–20], increase the total emission of volatile organic compounds (VOCs) [21–23] and lead to a poor fruit color development [24].

The Intergovernmental Panel on Climate Change (IPCC) currently estimates that global temperature has increased by 1 °C compared to pre-industrial levels, and in order to limit temperature rising to 1.5 °C greenhouse gas emissions have to reach net zero by 2050 (> 50% probability) [25], being this the most optimistic scenario (SSP1-1.9). A more plausible scenario (SSP1-2.6) indicates the world can limit temperature rise to 2 °C, while stabilizing it in 1.8 °C by the end of the century (> 67% probability), assuming net zero by 2070 [25, 26]. As such, a temperature rise under the current IPCC scenarios that might affect fruit traits is not just possible, but highly likely. Therefore, because of the importance of traits shaping the interactions between fruits and frugivores [27], trait alterations that affect the way frugivores recognize ripe fruits might also have an impact on seed dispersal networks [28]. The dispersion of seeds by animals plays an important role on the structure, biodiversity, and maintenance of natural ecosystems dynamics [29–33], hence dramatic changes on it could threaten ecosystem functioning, particularly in tropical regions where seed dispersal by animals is more common [34].

For example, warming may drive higher rates of scent emission from fruits [23], which could translate into a stronger chemical signal if relevant secondary metabolites are not a limiting factor [35], or to a reduction of the signal if it leads to faster depletion of synthesized chemicals. Such a scenario would be particularly relevant for systems like the one at focus – Madagascar – where fruit scent has been shown to drive animal fruit selection [11, 36]. Color, is also strongly associated with fruit selection [12, 37]; and as well as scent, it has been linked to nutrient content [11, 36–39]. Finally, fruit morphological traits like size and hardness [40] are recognized as limitations for frugivores when selecting fruits, (i.e. birds cannot manipulate nor eat fruits larger than their gape width) [41–43]. All these, while hard to predict, can cause major disruptions to existing dispersal networks by (a) directly altering signals like scent and color or their link with fruit quality, thus reducing animal foraging efficiency; (b) differentially changing the preference of fruit assemblages, driving an increase in dispersal of some species at the expense of others [13]; (c) reducing seed dispersal effectiveness [44, 45] by causing animals to consume more immature fruits, or by (d) changing interactions (e.g. rendering fruits smaller and more available to birds), thus causing a shift in the dispersal kernel of some species [42, 43].

As such, quantitatively, this effect can manifest in different degrees and ways in different systems where ecological networks (e.g. specialized vs. generalized) and sensory redundancy differ. But whether or not this is at all a risk hinges on two assumptions: that fruit traits drive fruit selection, and that they are affected by temperature. While the first is well established (see above), despite its importance, the question of how global warming might affect wild fruit traits has been widely overlooked. Regarding wild populations, previous research has focused on other plant traits (height, leaf area, seed-mass) and how they are affected by different environmental drivers (nitrogen deposition, precipitation) [46, 47] and on the geographic distribution of traits based on environmental variables [48]. Whereas studies on fruit traits have had solely an anthropogenic interest, in the sense they have only looked into cultivated species to evaluate quality parameters according to consumer and market preferences [17, 49, 50]. Regarding species interactions significant more attention has been given to the question of how temperature rise might affect flower traits and downstream flower-pollinators networks, indicating worrying trends which may or may not be similar to seed-dispersal networks [51, 52].

Under this framework, the goal of the current study was to evaluate, as a first proof of concept on a case study of five Malagasy plant species, the potential effect of temperature change on fruit functional traits of wild species. We used the elevation-for-temperature approach, in which elevational gradients provide a steady gradual temperature change of approximately − 0.55 °C for every 100 m upslope [53], while allowing the comparison of individuals belonging to a genetically continuous population [54], thus avoiding conflating genetically-determined phenotypic differences with variance driven by edaphic factors. This method has shown to be useful to predict how plants and other organisms might respond to global warming [55–58]. We analyzed fruit traits across a single slope in the montane primary forest of Mangevo, Madagascar, which compromises a continuous gradient of 520 m, providing an estimated temperature difference of 2.6 °C, from the lowest to the highest point, corresponding to end of the century IPCC projections and in line with projections for tropical regions [59]. Madagascar is a biodiversity hotspot where many local (often endemic) plants rely on the dispersal services of often endemic animals [60, 61] and it has already been used as a model system on animal-plant interactions in several studies [11, 12, 14, 38, 62]. We quantified five functional traits and focused on those that have been shown to be strongly associated with fruit selection and preference among frugivores in this or comparable systems: scent, color, size, hardness and sugar content. In short, fruit scent is increasingly recognized as a driver of fruit selection [63], specifically in this system it was observed to be associated with frugivore behavior [11] and be predictive of fruit quality (sugar content) [11, 36, 64]. Fruit color functions as a detection and selection signal to many frugivores [37, 65, 66], and particularly in this system has been observed to have evolved in response to frugivore visual systems [12]. Fruit size is a major driver of fruit selection in similar systems and beyond [14, 67], particularly birds who tend not to feed on fruits/seeds larger than their gape width [41, 43]. Fruit hardness has shown to be under an independent selection of mechanical constraints [14] and be associated with fruit selection in similar systems [40]. Finally, sugar is a major macronutrient sought after by frugivores and variation among fruits in sugar content has shown to affect fruit selection [68–70].

## Methods

### Model system and sample analyzes

**Fig. 1:**
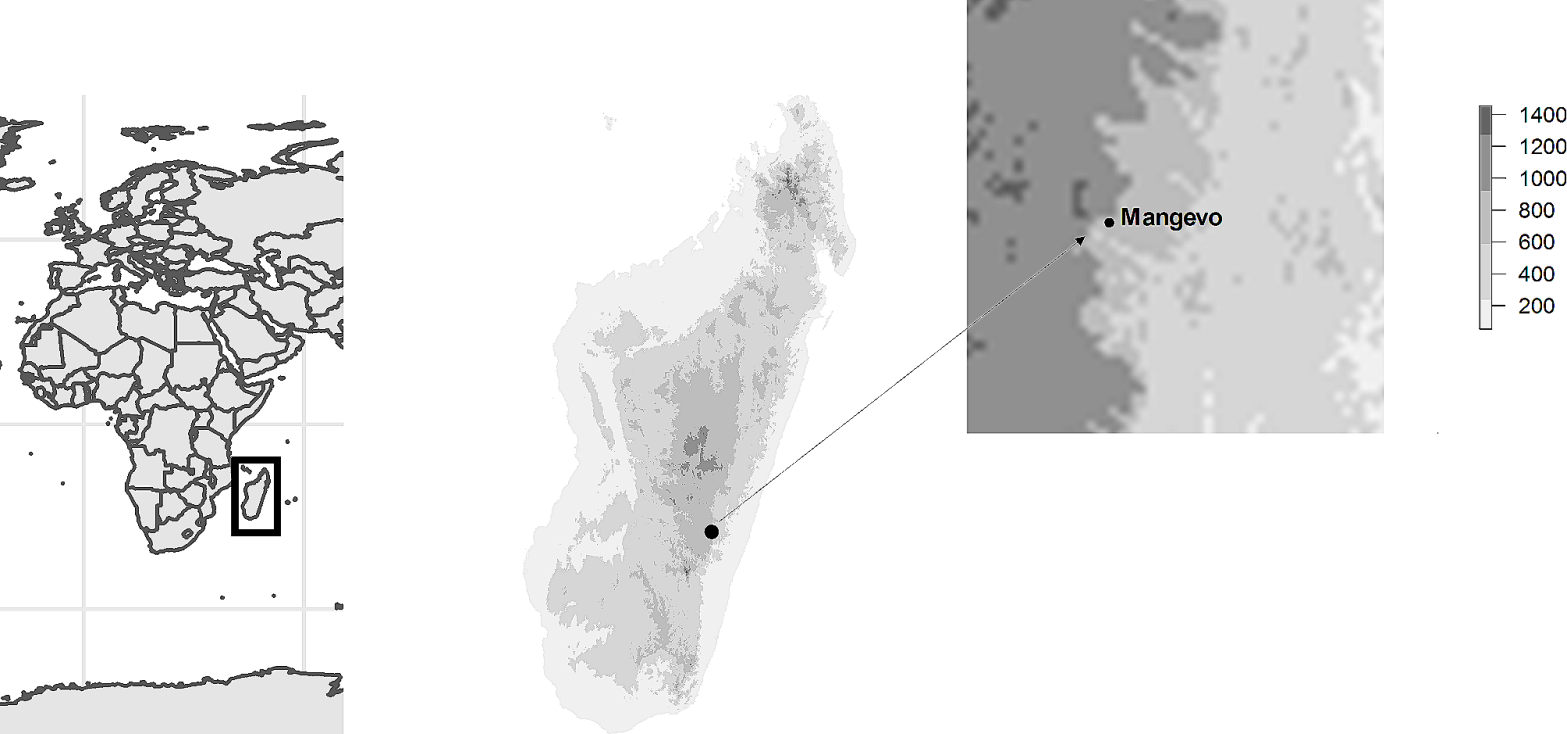
Map of Madagascar, showing Mangevo coordinates and elevational gradient

Fruit traits were analyzed directly on the field between May and June 2022, in the montane rainforest of Mangevo, a protected area that has never been logged, in Ranomafana National Park, eastern Madagascar (Fig. 1). We covered a single continuous forested slope from 580 to 1100 m above sea level (masl), which provided an estimated temperature difference of 2.6 °C. Across the elevational gradient, forest canopy closure was not significantly different [71] and precipitation was homogeneous.

Five species were selected based on the availability of ripe fruits across the gradient and their being at a reachable distance (max 4 m): *Ficus botryoides*, species with a lemur-specialist dispersion syndrome [11] and *Ficus politoria*, *Psychotria* sp1, *Psychotria* sp2, and *Pittosporum verticillatum*, all primarily dispersed by birds with some dispersal by lemurs [36]. The species sampled belonged to a single functional population, in which pollinators and frugivores can travel freely across the gradient [72–75]. All species are insect pollinated. Given that samples were all from a continuous community along a single slope, where seed dispersers and pollinators populations are also continuous on a relatively short distance that is very minor compared to disperser movement range, there is no reason to assume that genetic factors alone would generate a trait value gradient. Two to eight fruits were obtained from an individual plant and pooled together as a single sample (Table 1). Plants were identified on the field by a local expert: Pela August (technician at Ranomafana National Park), and no plant material was exported. Fruits recognized as ripe by their softening when mature, presence of mature seeds and their specific change in color. Fruits were brought within 3 h to the field processing station to measure scent, color, size, hardness and sugar content, in this order. The fruits are not suspected to systematically differ in their time on the maturation curve, which could in theory lead to a trait gradient if e.g. fruits mature earlier in lower elevations. This is because (a) all species had relatively low synchrony and hence crop of mature fruits at any given moment, which leads to a relatively quick removal by animals; and (b) we found very low correlation between fruit traits within species (mean *r* = 0.12), negating the possibility that some fruits were significantly more ripe than others.

**Table 1:** Description of number of samples analyzed for each species

Species	Number of individuals	Number of samples	Gradient (masl)	Temperature variance approximation (°C)
*Ficus botryoides*	11	37	627–903	1.5
*Ficus politoria*	22	38	627–1069	2.4
*Psychotria* sp 1	24	70	597–1072	2.6
*Psychotria* sp 2	24	54	628–918	1.6
*Pittosporum verticillatum*	13	37	680–993	1.7

### Analysis fruit traits

#### Scent

Fruit scent was sampled following a similar methodology to [11]. Fruits were placed in sampling bags of 40 cm (Toppits oven bags, Toppits). The bags were tightly closed with a zip tie at one end and on the other a Teflon tube with a chromatoprobe was mounted and carefully tighten. The chromatoprobes contained trapped in layers of glass wool 1.5 mg of Tenax, 1.5 mg of Carbotrap and 1.5 mg of Carbosieve III (all Sigma Aldrich). The samples were left to rest in the chamber for 20 min, afterwards the air was pumped for 10 min at 200mL/min. The probes were stored in 2 mL glass vials sealed with a Teflon cap and placed in at -32 °C upon return to the field station and until analysis. Controls to identify ambient contaminants were collected following the same procedure with empty bags.

##### Chemical analyses

Samples were analyzed using Shimadzu GCMS-QP2020 NX equipped with an TD30-R thermo desorption unit and a Shimadzu SH-RXI-5MS low polarity phase capillary column (SH-RXI-5MS, 30 m, 0.25 mm diameter). Samples were introduced to the thermal desorption unit and the tube heating started at 35 °C until it reached 300 °C. a Tenax liner was cooled to -20 °C. After the transfer to the liner, it was heated up until 230 °C. All samples were introduced into the system spitless, with the exception of *Pi.verticillatum*, for which split was set to 1:60 due to a high VOC signature that otherwise overloaded the column and resulted in broad unquantifiable peaks. As such, quantitative measures of fruit scent in this study are comparable within but not among all species. Initial oven temperature was 40 °C. This temperature was maintained for 1 min, then it was increased by 10 °C/min until it reached 280 °C, where it was held for 8 min. The MS transfer line temperature was set to 250 °C and MS source temperature was set to 230 °C. The MS operated at electron ionization mode and scanned between 30 and 400 m/z.

Chromatograms obtained were analyzed in AMDIS 2.7. VOCs were identified on the basis of their mass spectra by comparing them with the NIST11 mass spectra library and their retention index, which was calculated using a *n-*alkane reference mixture. Known contaminants (e.g. siloxanes) were fully removed from the dataset, whereas for genuine plant VOCs which were identified in both the controls and samples, we calculated the mean amount in the controls as a proxy of the baseline contamination level, and subtracted this amount from all samples. Peak area of all compounds was then divided by the respective number of fruits in each sample to obtain a standardized measure per functional unit (fruit).

VOCs were further classified to seven chemical classes based on functional or biosynthetic groups: alcohols, terpenes (including all monoterpenes, sesquiterpenes and derivates such as linalool), aldehydes, esters, aromatic (compounds with at least one aromatic ring), alkanes, and nitrogen containing compounds. The total amount of each class in each sample was calculated as the sum of all peaks belonging to the class.

#### Color

Fruits were photographed using a stand-mounted digital camera (Canon EOS rebel t3i, Canon Inc., Tokyo Japan) with a 18–55 mm lens (Canon EF-S 18–55 mm), at a focal length of 55 mm with no flash. The camera was positioned 40 cm directly in front of the fruits. The shots were intentionally underexposed to avoid the loss of data by overexposing [76, 77]. To calibrate and standardize the photos we used a color chart (ColorChecker Classic, X-Rite, Grand Rapids, MI, USA) [78]. The fruits were placed in front of the chart. Because the chart and the fruits could not be focused on at the same time, a first shot focusing the fruits was taken and immediately after a second shot was taken focusing the chart, this assured the pictures had the same light conditions and camera settings [77, 79]. All files were exported in the RAW format, because it is linear and displays a wider variety of colors [77, 79] .

The photographs were processed in ImageJ v1.8.0_172 [78] using the micaToolbox plugin [80]. Each photo was converted into a multispectral image, using the 5% and 95% reflectance standards and the regions of interest were selected [77, 78]. Because the camera was not UV sensitive the analyses were restricted to visible spectrum (400–700 nm) and the percentage values of the red, green and blue (RGB) color channels were extracted from each photograph.

#### Size

The size of each fruit was determined by measuring the dimensions along cardinal directions (length, width and depth) using sliding calipers. Afterwards, the fruit volume was stablished using the ellipsoid formula: $$V=\raisebox{1ex}{$4$}\!\left/ \!\raisebox{-1ex}{$3$}\right.*\pi abc$$ [14].

#### Hardness

Hardness was defined as skin puncture resistance in $kg/{mm}^{2}$ and measured using a hand-held durometer (Shimpo MX) [14, 40, 81]. Fruit hardness was not measured for the species *P. verticillatum*, because of the dehiscent nature of this species when ripen.

#### Sugar content

Consistently for each species, based on the size of the fruits, between 0.5 and 2 gr were macerated with 1–2 mL of water (*F.botryoides* 2gr with 2mL, *F. politoria* 1gr with 1mL, *Psychotria* sp 1 0.5gr with 1mL and *Psychotria* sp 2 and *P. verticillatum* 1gr with 2 mL), until a homogenous solution was obtained. Four drops of the solution were used to measure the sugar content with a refractometer (MASTER-AGRI, Atago). Refractometers have demonstrated to be good instruments to measure the total content of sugar in fruits [82, 83]. Results are expressed in Brix/TSS (total soluble solids) and represent the percentage of sugar present in a fruit. Given that the amount of fruit flesh and water added was standardized within species, these results allow a reliable comparison of the relative concentration of sugar *within species and only within our dataset* but are not valid for across-species comparisons or for estimation of absolute amounts.

### Statistical analyses

Multiple samples from the same individual tree were pooled by averaging values, making individual tree the basic datapoint in all analyses (Table 1). To analyze the effect of temperature on each fruit trait, we applied two similar but complementary approaches. The first was generalized linear models (GLMs) conducted for each species separately in which a trait was set as the response variable: total amount of VOCs, total amount of each VOC class, percentage RGB color channels, hardness $$\left(kg/{mm}^{2}\right)$$, size ($${mm}^{3})$$ and sugar content (Bx°). Elevation in m was the sole predictor (fixed) factor. We then applied a Hommel correction for multiple testing. For all models, we verified the normal distribution of the random effect as well as the normality and homogeneity of the residuals using histograms, quantile-quantile plots, and plotting the fitted vs. the residuals. In the cases where assumptions were not met, the data was log-transformed before running the model. These analyses were conducted because we did not have a clear directional hypothesis (e.g. that higher elevations are associated with redder fruits). In addition, we also ran generalized linear mixed models (GLMMs) with a gaussian error structure in which each of the predictors mentioned above was a sole predictor, elevation was a fixed factor, and species as a random intercept factor. This analysis was set to test if trends across species are similar or not, although it should be noted that the use of species as a random factor violates the assumption of full randomness. *P*-values were calculated by comparing the full models with their respective null models, which did not include elevation in a likelihood ratio test. All analyses were performed in *R* studio 4.1.2 with the package lme4 [84].

## Results

**Fig. 2:**
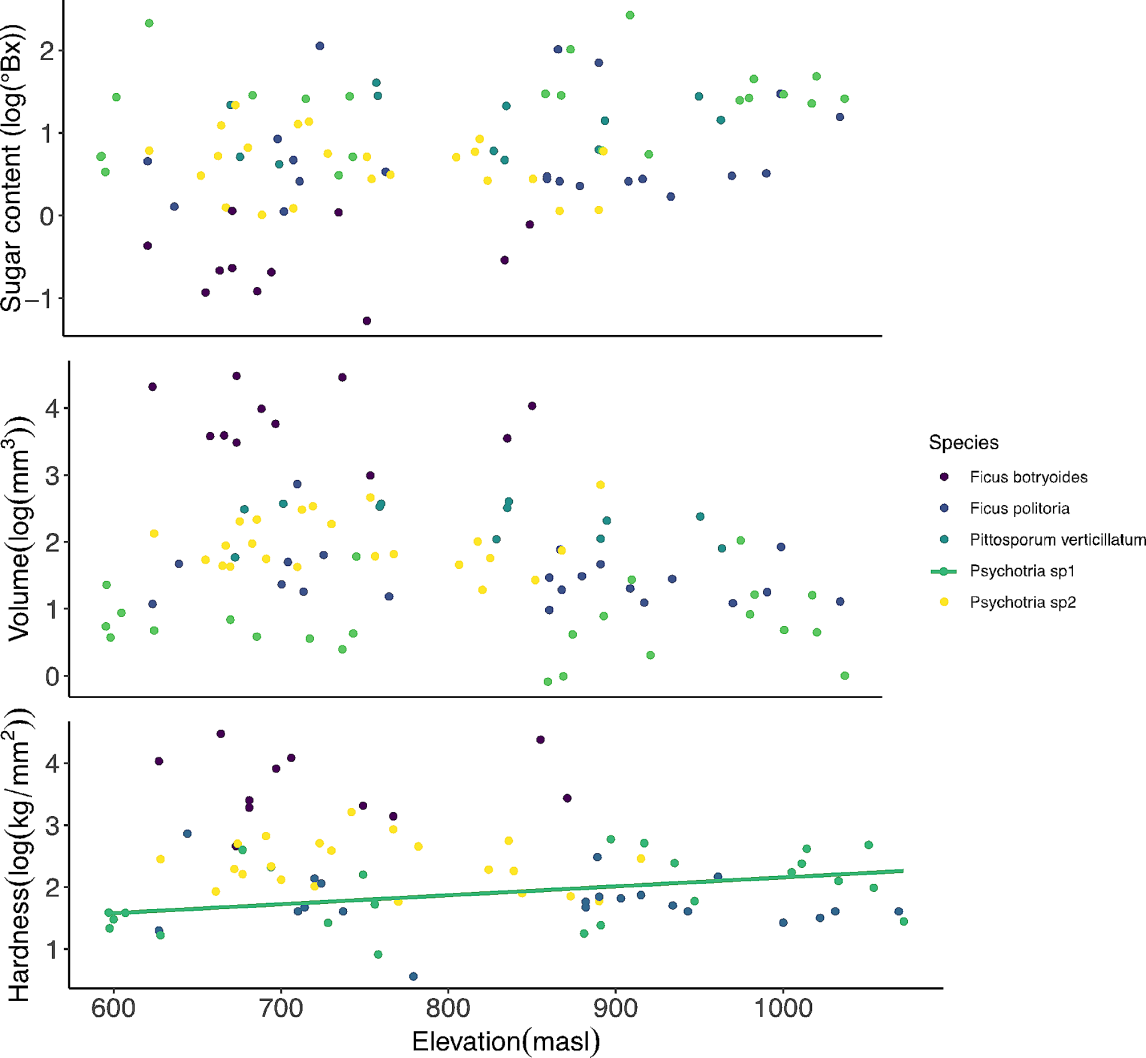
Relationship between elevation (masl) and fruits sugar content; size; hardness, green slope with *Psychotria* sp1 (*p = 0.03*), in all five species. Sugar content was measured in °Brix with a refractometer by macerating 0.5-2gr with 1-2mL of water (depending on the species). Size was calculated based on fruit dimensions assuming ellipsoid shape in ${mm}^{3}$. Hardness was defined as skin puncture resistance in $kg/{mm}^{2}$. All data log transformed to comply with the statistical test assumptions

**Fig. 3:**
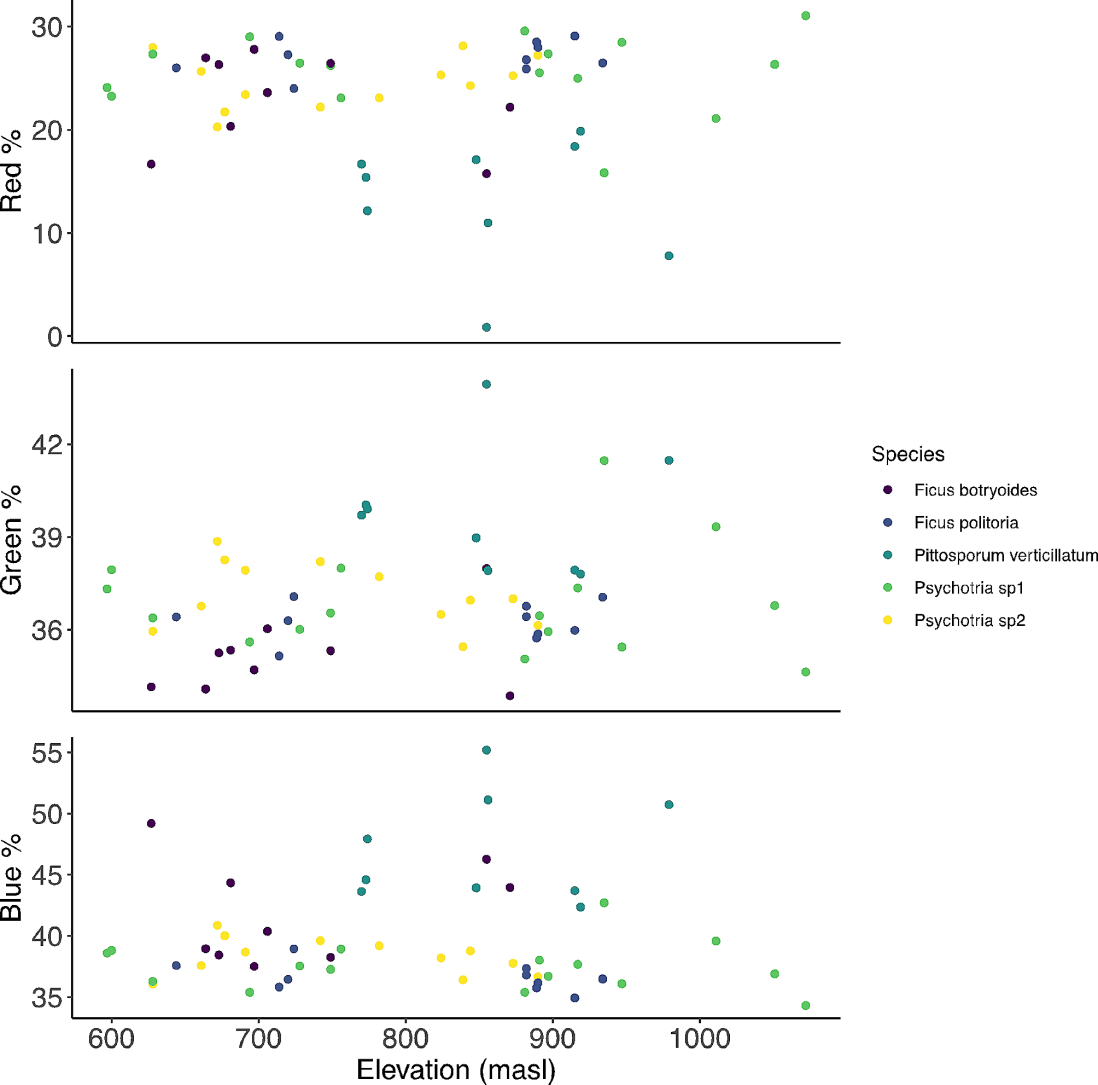
Relationship between elevation (masl) and red%; green%; blue% in all five species. The RGB percentage values were extracted in ImageJ software using the MicaToolbox plugin. All photographs were standardized with a color chart (ColorChecker Classic, X-Rite, Grand Rapids, MI, USA)

**Fig. 4:**
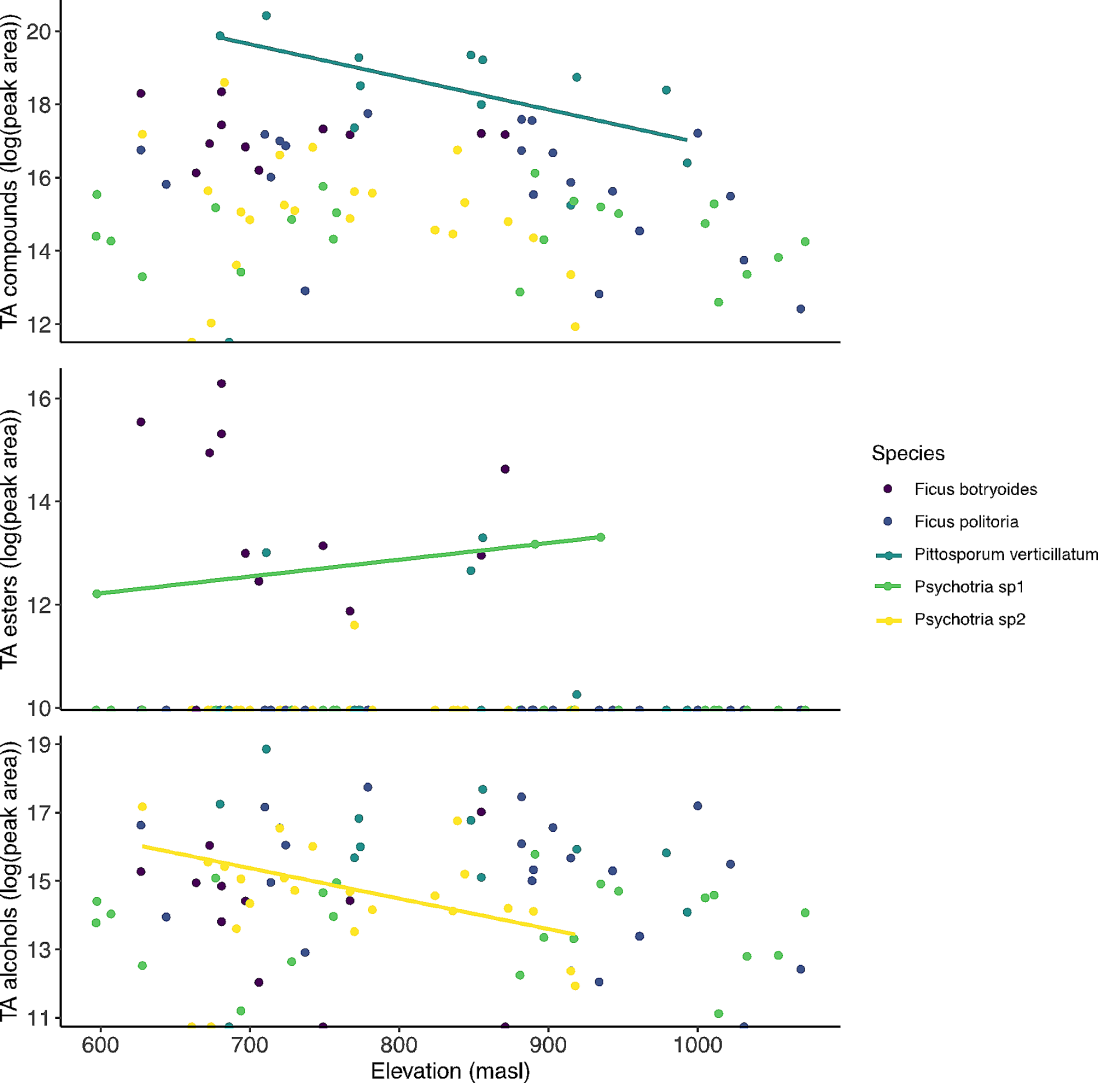
Relationship between elevation (masl) and total amount (TA) of volatile organic compounds, light blue slope with *P. verticillatum* (*p* = 0.036); volatile esters, green slope with *Psychotria* sp1 (*p =* 0.039), and volatile alcohols, yellow slope with *Psychotria* sp2 (*p =* 0.029), in all five species. Total amount of VOCs was defined as the summatory of the area of all peaks in all samples and total amount of each class of compound was defined as the summatory of the area of all peaks corresponding to each class in all samples. All data log transformed to comply with the statistical test assumptions

The linear models showed that after correction for multiple testing, most traits did not change across the gradient within species ($p>0.05$), with some exceptions: hardness and esters in *Psychotria* sp1 had a positive correlation with elevation ($p=0.03; p = 0.039$, respectively), whereas the total amount of VOCs in *P. verticillatum* and total amount of volatile alcohols in *Psychotria* sp2 had a negative correlation with elevation (*p* = 0.036; *p* = 0.029 respectively) (Table S1). All mixed models examining fruit traits as a function of elevation were not significantly different from their respective null models $\left({[X}^{2}=0 \left(1\right), P=1\right],$in all models), indicating no consistent effect of elevation on any trait across the gradient (sugar content, size and hardness: Fig. 2; RGB color channels: Fig. 3; scent: Fig. 4**)**.

## Discussion

The objective of our study was to use the elevation-for-temperature approach to estimate, for the first time to our knowledge, whether temperature variance may affect a set of wild fruit functional traits that are known to be relevant for seed-dispersal networks. We did so on a case study of five Malagasy plant species sampled along a continuous forested slope between 597 and 1072 masl, corresponding to a temperature variance between 1.5 and 2.6 °C [53] – in line with the most likely IPCC scenarios to the end of the century [25, 26] and in line with predicted temperature increase in the tropics [59].

### Scent

Scent is possibly the trait most likely to be affected by temperature change as it is comprised of dozens to hundreds of different VOCs and each can be affected differently by ambient temperature. In contrast to other traits, it can be affected directly by changing enzymatic efficiency or VOC volatilization from the fruit or indirectly (temperature changes growth conditions which lead to differential VOC synthesis). In our study *P. verticillatum* was the only species which showed a higher amount of total amount of VOCs at higher temperatures, whereas the total amount of VOCs of the other species did not change. This increase of VOCs at higher temperatures agrees with previous studies that argue the world will become a more scented place with global warming, as high temperatures enhance enzymatic activity of VOCs biosynthesis, decrease diffusion pathway resistance and increase volatility [21, 22, 35].

Nonetheless, existing data on mostly cultivated species also does not reveal a systematic pattern of increase in VOC emission with temperature [23, 50, 85]. This indicates that any effect of temperature on the total emission of VOCs may be subtle and possibly limited to the responses each compound class; therefore, the changes on the chemical profile of species probably depend on the specific compounds that constitute it. In our results, different classes of compounds showed different responses to the temperature gradient: while volatile alcohols and terpenes decreased at higher elevations in *Psychotria* sp 2 and *P verticillatum* respectively, esters of *Psychotria* sp 1 increased at higher elevations as well as aromatic compounds in *F. botryoides* (see Table S1). Although sample size of esters in *Psychotria* sp 1 was very small, previous results by [85] also showed an increase of this class of compound at higher elevations in cultivated bananas. We recognize the correlation of terpenes in *P. verticillatum* and aromatic compounds in *F. botryoides* were not statistically significant after correction for multiple testing. However, previous studies in flowers suggest these results could be genuine [21, 86], still further examination is required.

Future studies should aim to elucidate the effect of temperature on: (1) different species, especially those with a mammalian dispersal syndrome, as is in these species where scent plays a major role in fruit recognition [11, 87] and consequently in the ones that changes could cause major disturbances in ecosystems [88]; and (2) different compounds and classes of compounds, focusing attention on those that have shown to play a key role in fruit recognition by seed dispersers, as are esters or terpenoids [36, 63]. Changes in the VOCs profile of flowers have already shown to be detrimental in pollination networks, as they have altered fidelity and probability of pollinators recognition [21], highlighting the importance of scent in mutualistic networks.

### Color

Fruit color is sensible to temperature changes, because temperature can influence on the synthesis of pigments (e.g.: anthocyanins and carotenoids) [24] and for most species increases in temperature have shown to down-regulate the genes involved in these pathways [24, 89]. Accordingly, global warming has the potential to lead to an impairment in fruit coloration that can negatively alter the way frugivores identify ripe fruits. In our study, none of the fruits evaluated presented a change in their color channels in response to the temperature gradient. Previous studies on cultivated species have shown that significant changes in fruit coloration usually occur at temperatures above 27ºC [90–92], therefore the species evaluated here most probably did not change because temperatures did not surpass 22ºC. Nonetheless, because other regions most likely will exceed 27ºC with global warming [93], it is important that future studies foresee the implications this could have for seed dispersal networks. Future works on color should ideally include fruit reflectance at the UV part of the spectrum. This is particularly relevant for birds and while requiring special instrumentation, it has been successfully demonstrated in this and other model systems [94].

Another environmental variable that regulates fruit coloration is solar UV radiation and contrary to temperature, it has been observed that UV exposure increases the synthesis of pigments [18, 81, 82]. UV-radiation increases with altitude, up to 10% for every 1000 m, and consistently several studies on cultivated species show that fruits at higher elevations have a darker as well as a more intense color [18, 82, 95]. However, this induced accumulation of pigments by UV-radiation is limited by increased temperatures [91, 96]. The species evaluated here, were not affected by UV-radiation because forest canopy was homogenous across the elevational gradient [71].

### Size

Temperature affects fruit size by accelerating (high temperatures) or slowing down (low temperatures) the ripening process [18]. A slower ripening leads to a prolonged accumulation of photo-assimilates that ultimately leads to fruits being bigger [81, 97]. Fruit size did not change for any of the species evaluated here. This is most probably because the temperature variance was not enough to cause changes in the photo-assimilates accumulation rate of the species examined.

### Hardness

Previous studies on cultivated species indicate temperature affects fruit hardness; however, the directionality (i.e., whether fruits become softer or harder) by an increase in temperature varies across species [97–99]. In this study, only one species *Psychotria* sp1 increased its hardness at lower temperatures (higher elevations), whereas the other species did not change.

To explain the mixed responses of fruit hardness towards temperature, authors have proposed that this trait strongly depends on cell wall composition as well as calcium and starch concentration, intrinsic physiological characteristics related to fruit softening [18]. Therefore, more research is needed to better elucidate the ways in which global warming might affect fruit hardness, more importantly because further environmental changes are expected with global warming. For instance, changes in rainfall regimes, which can subsequently alter UV-radiation and relative humidity through changes in cloud patterns [100, 101]; factors that have also shown to influence in fruit hardness: while UV has shown to increase it, relative humidity to decrease it [16, 18, 81].

### Sugar content

Temperature at which a fruit develops influences the amount of sugar content [18]. According to previous studies, a mild increase in temperature can promote the translocation of sugars to fruits, hence increasing sugar content [97]. Nonetheless, when the increase of temperature surpasses the normal range of species, it causes a loss of carbohydrates by an acceleration in respiration [18, 81, 99]. It is therefore, of high concern that fruits become less sugary as a consequence of global warming, as it could cause a deficit in the energy frugivores obtain when foraging. The fruits studied here did not change their fruit sugar content in response to the temperature gradient, probably because it was inside their normal range. However, other regions might experience increases in temperature that are outside the tolerant ranges of populations [102], it is then important that future studies bring attention to changes in fruit sugar content in response to global warming.

## Synthesis, caveats, and conclusions

Under the temperature gradient examined, which corresponds to IPCC predictions for the end of the century, most of the traits in the five species included did not show significant changes. This indicates prima facia that fruit functional traits, measured in the model system studied here, may not be strongly, or at least systematically, affected by anticipated temperature rise by the end of the century and that most likely the frugivores present will be able to keep on relying on these traits to successfully identify ripe fruits. However, this pattern may also be explained by other factors. A likely explanation is that the current projected change is inside the tolerant temperature range of the species examined, hence suggesting species are likely to maintain a similar expression of their functional traits. Nonetheless, while the projected temperature increase will not push the species studied here into an environment that substantially change their fruit phenotypes, further temperature rise may still lead to change, and individuals growing in higher or lower elevations will find themselves outside their optimal temperature range. This can lead to a shift in fruit traits, altering fidelity in which frugivores successfully identify ripe fruits and potentially affecting forest regeneration and structure.

Another important caveat is that the model system used here was limited to only five species. While significant in the sense that data on this question from the wild are all but absent, it is imperative for future studies to build on our results – expanding model species, traits, season variation, and elevation (or temperature directly) gradients – to assess whether these patterns are an exception or the rule.

Further, an advantage of the elevation-for-temperature approach is that it can assess the effect of temperature independent of many other factors such as rainfall. Yet global warming is likely to bring about many other changes in addition to mere temperature change: precipitation patterns, wind regimes, soil degradation and other factors which together may drive more significant and disruptive changes to fruit traits [103–105]. Along these lines, global warming may directly and indirectly change animal populations and behavior, with potential downstream effects for seed dispersal dynamics. Finally, global warming may affect fruit traits indirectly by altering pollination patterns.

It is important to note that abiotic conditions along an elevational gradient are not always restricted to temperature, and may include variation in rainfall patterns, soil quality, etc., yet given the relatively high homogeneity along the gradient studied here [71], these factors are unlikely to have affected the results. Therefore, we can infer that nor temperature, which did change across the elevational gradient, nor other environmental factors strongly affected the fruit traits in this system.

As such, our results should be taken only as an indication that global warming is not likely to dramatically and immediately change fruit phenotypes across all traits and in all fleshy fruits. By no means do they indicate that global warming will not change fruit traits and downstream animal-plant interactions and seed dispersal networks. Indeed, the rich body of literature on cultivated species indicates that effects are not uniform and not always linear. Rather, they indicate that the effects are complex and will differ across species and systems. Therefore, we highlight the need for more nuanced analyses on wild species across systems, that contribute to test predictions on the possible downstream effects that trait alterations could cause on the ecosystem functions sustained by seed dispersal interactions. We hope that our work will serve as a starting point in this endeavor so that species and systems which are vulnerable to a rapidly changing world are identified sooner rather than later.

### Electronic supplementary material

Below is the link to the electronic supplementary material.

Supplementary Material 1

Supplementary Material 2

## Data Availability

All raw data is available as online supplementary material.

## Abbreviations

IPCC: Intergovernmental Panel on Climate Change
RGB: Red, green, blue
VOCs: Volatile organic compounds
