# Supplementary material for: Effects of temperature gradient on functional fruit traits: an elevation-for-temperature approach
Source: BMC Ecol Evol. 2024 Jul 9;24:94. doi: 10.1186/s12862-024-02271-w (PMC11232184; doi:10.1186/s12862-024-02271-w)
Supplement: Supplementary file 1 — Supplementary Material 1 [file 12862_2024_2271_MOESM1_ESM.docx]

**Effects of temperature gradient on functional fruit traits: an elevation-for-temperature approach**

**Laura Gómez-Devia & Omer Nevo**

lgomez-devia@imedea.uib-csic.es; +34625850968

**Table 1S.**

*p* values from Generalized Linear Models: elevation ⁓ total amount of the different classes of compounds identified in the five species examined. Total amount of each class of compound was defined as the summatory of the area of all peaks corresponding to each class in all samples. N/A: compound class not found in species, *p*-bef = before correction for multiple testing; *p*-aft = after correction for multiple testing.

| Species | Alcohols | | |  | Terpenes | | | |  | Esters | | |  | Aromatic compounds | | | |
| --- | --- | --- | --- | --- | --- | --- | --- | --- | --- | --- | --- | --- | --- | --- | --- | --- | --- |
|  | **F(dfs)** | ***p-*bef** | ***p-*aft** |  | | **F(dfs)** | ***p-*bef** | ***p-*aft** |  | **F(dfs)** | ***p-*bef** | ***p-*aft** |  | **F(dfs)** | ***p-*bef** | ***p-*aft** |  |
| *Ficus botryoides* | $F_{1-7}=0.87$ | 0.38 | 0.9 |  | | $F_{1-9}=0.19$ | 0.67 | 1 |  | $F_{1-8}=1.75$ | 0.23 | 0.46 |  | $F_{1-5}=9.48$ | **0.028*** | 0.112 |  |
| *Ficus politoria* | $F_{1-18}=1.13$ | 0.30 | 0.9 |  | | $F_{1-1}=0.13$ | 0.78 | 1 |  | N/A | N/A | N/A |  | N/A | N/A | N/A |  |
| *Psychotria* sp 1 | $F_{1-19}=0.70$ | 0.79 | 0.9 |  | | $F_{1-2}=0.51$ | 0.55 | 1 |  | $F_{1-1}=2293$ | **0.013*** | **0.039*** |  | $F_{1-1}=0.04$ | 0.87 | 1 |  |
| *Psychotria* sp 2 | $F_{1-19}=9.72$ | **0.006*** | **0.029*** |  | | N/A | N/A | N/A |  | N/A | N/A | N/A |  | $F_{1-1}=0.76$ | 0.54 | 1 |  |
| *Pittosporum verticillatum* | $F_{1-10}=6.53$ | **0.029*** | 0.11 |  | | $F_{1-10}=5.62$ | **0.039*** | 0.156 |  | $F_{1-2}=1.38$ | 0.36 | 0.46 |  | $F_{1-1}=0.72$ | 0.55 | 1 |  |

| Species | Alkanes | | |  | Nitrogen compounds | | |  | Aldehydes | | |
| --- | --- | --- | --- | --- | --- | --- | --- | --- | --- | --- | --- |
|  | **F(dfs)** | ***p-*bef** | ***p-*aft** |  | **F(dfs)** | ***p-*bef** | ***p-*aft** |  | **F(dfs)** | ***p-*bef** | ***p-*aft** |
| *F. botryoides* | $F_{1-7}=1.70$ | 0.23 | 1 |  | $F_{1-5}=0.40$ | 0.55 | 1 |  | $F_{1-4}=0.56$ | 0.49 | 1 |
| *F. politoria* | $F_{1-10}=1.16$ | 0.31 | 1 |  | $F_{1-11}=0.29$ | 0.87 | 1 |  | $F_{1-8}=0.11$ | 0.75 | 1 |
| *Psychotria* sp 1 | $F_{1-17}=0.04$ | 0.84 | 1 |  | $F_{1-13}=0.23$ | 0.64 | 1 |  | $F_{1-5}=0.34$ | 0.59 | 1 |
| *Psychotria* sp 2 | $F_{1-10}=1.04$ | 0.33 | 1 |  | $F_{1-6}=0.24$ | 0.64 | 1 |  | $F_{1-6}=0.74$ | 0.73 | 1 |
| *P. verticillatum* | $F_{1-7}=0.24$ | 0.64 | 1 |  | $F_{1-4}=2.46$ | 0.19 | 0.95 |  | N/A | N/A | N/A |
